# Genome position specific priors for genomic prediction

**DOI:** 10.1186/1471-2164-13-543

**Published:** 2012-10-10

**Authors:** Rasmus Froberg Brøndum, Guosheng Su, Mogens Sandø Lund, Philip J Bowman, Michael E Goddard, Benjamin J Hayes

**Affiliations:** 1 Centre for Quantitative Genetics and Genomics, Department of Molecular Biology and Genetics, Faculty of Science and Technology, Aarhus University, Tjele, 8830, Denmark; 2 Biosciences Research Division, Department of Primary Industries Victoria, Bundoora, 3083, Australia; 3 Land and Food Resources, University of Melbourne, Parkville, 3072, Australia; 4 Dairy Futures Cooperative Research Centre, Bundoora, Victoria, 3083, Australia; 5 La Trobe University, Bundoora, Victoria, 3086, Australia

**Keywords:** Genomic prediction combined populations, Genomic location, Bayesian prediction

## Abstract

**Background:**

The accuracy of genomic prediction is highly dependent on the size of the reference population. For small populations, including information from other populations could improve this accuracy. The usual strategy is to pool data from different populations; however, this has not proven as successful as hoped for with distantly related breeds. BayesRS is a novel approach to share information across populations for genomic predictions. The approach allows information to be captured even where the phase of SNP alleles and casuative mutation alleles are reversed across populations, or the actual casuative mutation is different between the populations but affects the same gene. Proportions of a four-distribution mixture for SNP effects in segments of fixed size along the genome are derived from one population and set as location specific prior proportions of distributions of SNP effects for the target population. The model was tested using dairy cattle populations of different breeds: 540 Australian Jersey bulls, 2297 Australian Holstein bulls and 5214 Nordic Holstein bulls. The traits studied were protein-, fat- and milk yield. Genotypic data was Illumina 777K SNPs, real or imputed.

**Results:**

Results showed an increase in accuracy of up to 3.5% for the Jersey population when using BayesRS with a prior derived from Australian Holstein compared to a model without location specific priors. The increase in accuracy was however lower than was achieved when reference populations were combined to estimate SNP effects, except in the case of fat yield. The small size of the Jersey validation set meant that these improvements in accuracy were not significant using a Hotelling-Williams t-test at the 5% level. An increase in accuracy of 1-2% for all traits was observed in the Australian Holstein population when using a prior derived from the Nordic Holstein population compared to using no prior information. These improvements were significant (P<0.05) using the Hotelling Williams t-test for protein- and fat yield.

**Conclusion:**

For some traits the method might be advantageous compared to pooling of reference data for distantly related populations, but further investigation is needed to confirm the results. For closely related populations the method does not perform better than pooling reference data. However, it does give an increased accuracy compared to analysis based on only one reference population, without an increased computational burden. The approach described here provides a general setup for inclusion of location specific priors: the approach could be used to include biological information in genomic predictions.

## Background

Genomic predictions are now widely used in dairy cattle breeding, and have been proposed for breeding of crops and prediction of disease risk in humans [1,2]. The accuracy of genomic estimated breeding values, depends on a number of factors, of which the size of the reference population used to estimate the marker effects is critical [3]. In dairy cattle, the reference population usually consists of progeny-tested bulls. In small populations, such as Australian Jersey, the number of progeny-tested bulls available for the reference is limited. For genetically related populations such as the European Holstein populations and Nordic Red Cattle populations previous studies show large benefits from pooling reference populations [4,5], but for more distantly related populations (e.g., Holstein and Jersey) this approach does not increase the accuracy to the same extent [6,7]. Previous studies based on Single Nucleotide Polymorphism (**SNP**) markers from the Illumina 50K SNP chip [8] have reported that distances between markers would be too large for high persistence of linkage disequilibrium (**LD**) phase across breeds, and accuracies of across breed prediction were zero [9,10]. With the new Illumina 777K chip, it is expected that distances between markers are small enough for successful genomic prediction using combined reference data from different dairy cattle populations, as the Quantitative Trait Loci (**QTL**)-SNP phase in such high density markers would be well preserved across breeds [3]. However, a recent study demonstrated only limited support for this hypothesis, with relatively small gains resulting from pooling Australian Holstein and Jersey data to improve the accuracy of Jersey genomic predictions [11]. This suggests that there are still differences in the patterns of LD between single markers and actual QTL across breeds, and thus pooling the data might dilute associations of markers with phenotypic traits.

In this study, we explore an alternative approach to pooling data across breeds. Previous studies have shown that some parts of the genome explain more variation than others. Assuming that the same causative mutations, or even the same gene regions but different causative mutations, act on traits of interest in different populations, it is expected that effects of chromosome regions on a trait could be consistent among populations, though the LD patterns between individual SNPs and QTLs could differ from one population to the other. At the extreme it was demonstrated that there was considerable overlap in gene regions affecting stature in humans and cattle [12]. The aim of this study was to first map the variation explained by small segments of the bovine genome for production traits in three dairy cattle populations, and compare this variation across the populations. In particular, we explored the effect of segment size on the correlation of the variances across populations. Subsequently this information was used as genomic location specific priors in a new method for predicting genomic estimated breeding values. Developing a model with location specific prior information will also allow for differentiation between e.g. coding and non-coding regions of the genome, or other kinds of biological information.

## Methods

### Data

The datasets used in the present study included 540 Australian Jersey bulls (**JER-AUS**), 2297 Australian Holstein bulls (**HOL-AUS**) and5214 Nordic (Danish, Swedish or Finnish) Holstein bulls (**HOL-NOR**) (Table 1). Phenotypic data was daughter trait deviations (**DTD**) for the Australian bulls and deregressed proofs (**DRP**) for the Nordic bulls. DTDs were given in kilograms, whereas the DRPs are standardized indices. The traits selected for the study were: protein yield, fat yield and milk yield, as these traits have the most data in the populations.

**Table 1:** Data overview

**Breed**	**N ref**	**N val**	**N total**	**N markers**	**Phenotype**
JER-AUS	454	86	**540**	465542	DTD
HOL-AUS	1897	360	**2197**	465542	DTD
HOL-NOR	**3047**	2167	5214	465542	DRP

The populations used for the analysis, the number of animals in the reference (N ref) and in the validation set (N val), total number of animals (N total), number of markers (N markers) and type of response variable (Phenotype) used in the prediction models. The sets of animals used for estimation of segment variances are highlighted in bold.

Genotypic data was a mixture of true and imputed SNP markers from the Illumina 777K SNP chip. For HOL-AUS there were 843 Holstein heifers genotyped on the 777K SNP chip as well as 93 key ancestor bulls. For JER-AUS 93 key ancestor bulls were genotyped for the 777K SNP chip. Quality control steps included removing SNPs with very low minor allele frequencies, ambiguous or undefined map positions, and no heterozygote genotypes. For full details see [11]. These animals were used as reference to impute the high density genotypes for the remaining 2204 Holstein and 447 Jersey bulls which were genotyped with the 50k chip.

For HOL-NOR 557 bulls from the EuroGenomics project [4] were genotyped using the 777K chip and these bulls were used as reference to impute the 777K markers for the bulls genotyped with the Illumina 50K SNP chip. After imputation LD of each marker with the previous one in the assembly was inspected. If two adjacent markers were in complete LD one of the markers was deleted, so that r^2^ of any pair of adjacent markers was less than one. The marker data was further edited by deleting markers with a minor allele frequency less than 0.01.

Imputation was done using Beagle [13] in all three populations. Since the purpose was to compare segments across populations and use this information for genomic prediction, the SNP datasets were further edited to only keep the markers that were in common across the populations. After data editing 465,542 markers remained for analysis.

Each of the datasets was split into a reference and validation set (Table 1) to allow for cross validation of the accuracy of DGV. In HOL-NOR the bulls were separated by birth date before or after 2001-10-01, and in JER-AUS and HOL-AUS the bulls were split by onset of progeny test before or after 2007. In both cases the younger animals were assigned to the validation set. This cross validation strategy was chosen as the resulting accuracy is the most meaningful in the context in which the genomic predictions will be used: in the dairy industry. Here reference sets of older bulls are used to predict the DGV for young bulls which are then selected for use based on these DGV. In the Australian data all of the available data was used to estimate segment variances to maximize the data. In the Nordic dataset only the reference set was used to estimate segment variances.

All genotypic and phenotypic data was obtained from pre-existing routine genetic evaluation data for the dairy cattle populations and required no ethical approval.

### Estimation of genetic variances explained by different segments

Genetic variance attributed to each segment was estimated from effects of the SNPs in the segment. Prediction of single SNP marker effects was carried out using BayesR [11]. The statistical model was:

(1) y=μ1+Wg+a+e

Where **y** is the vector of DTD or DRP, μ is the mean, **a** is the vector of residual polygenic effects, **e** is the vector of the residual errors, **W** is the incidence matrix of SNP genotype coefficients, and **g** is the vector of SNP allele substitution effects. Let **X** be a genotype matrix coded as 0/1/2, for respectively homozygote first allele, heterozygote and homozygote second allele, and let p_j_ be the frequency of the second allele at locus j. Assuming Hardy-Weinberg equilibrium, entries of **W** are then obtained by centering and standardizing entries of **X** to:

(2) $$w_{\mathrm{ij}}=\frac{x_{\mathrm{ij}}-2p_{j}}{\sqrt{2p_{j}\left(1-p_{j}\right)}}$$

Prior distributions for the parameters are given as:

(3) $$g_{j}|\sigma_{j}^{2}\sim N\left(0,\sigma_{j}^{2}\right)\sigma_{j}^{2}=\{\begin{array}{c} 0*\sigma_{g}^{2}\;\text{with}\;\text{probability}\;\pi_{0}\\ 0.0001*\sigma_{g}^{2}\;\text{with}\;\text{probability}\;\pi_{1}\\ 0.001*\sigma_{g}^{2}\;\text{with}\;\text{probability}\;\pi_{2}\\ 0.01*\sigma_{g}^{2}\;\text{with}\;\text{probability}\;\pi_{3} \end{array}\;\sigma_{g}^{2}=r_{y}^{2}*\text{Var}\left(y\right)$$

(4) $$\pi \sim \text{Dirichlet}\left(1,1,1,1\right)\;e\sim N\left(0,I\sigma_{e}^{2}\right)\;a\sim N\left(0,A\sigma_{a}^{2}\right)\;\sigma_{e}^{2},\sigma_{a}^{2},\mu \;\propto \;1$$

Where **A** is the additive relationship matrix, σ_a_^2^ is the variance of residual polygenic effects, and *r*_*y*_^2^ is the reliability of DRP/DTD. The four-distribution mixture chosen for the SNP effects, does not reflect any biological hypothesis, but was chosen to allow for easier mixing between SNPs with no effect and SNPs with effects of different sizes. The Dirichlet prior on the proportions of different SNP variances with all parameters set to one, is actually a uniform prior, but specifying it in this manner reflects the fact that the posterior distribution on the proportions follows a Dirichlet distribution with a pseudo count of 1 from each of the four distributions. The prior is not uninformative in any statistical sense since it states that all distributions have equal probabilities, but it adds very little information compared to the posterior, as the data gives information on almost half a million counts, and the prior only adds 4, see [11] for more detail.

To estimate the variance explained by each small chromosomal region, the entire set of SNPs was divided into segments of a fixed length (e.g. 100 markers each).The variance explained by segment s was calculated as

(5) $$Var\left(W_{s}g_{s}|data\right)$$

Where **W**_**s**_ is the sub-matrix of **W** corresponding to the SNPs in segment s, and **g**_**s**_ is the vector of estimated SNP marker effects for the same segment, i.e. the segment variance is the variance across individuals of the partial direct genetic values (DGVs, marker only estimated breeding values) belonging to segment s. Variances of the partial DGVs for all segments were calculated at each iteration in the Gibbs sampler, and the estimated segment variances were obtained as the posterior means. Segment variances were estimated for segment sizes of 10, 25, 50, 100, 250, 500, 1000, 2000 or 3000 SNPs and for entire chromosomes. The approach is similar to [14] where sliding windows of five consecutive SNPs are used to estimate the genetic variance of chromosomal regions. In our approach the windows are however not overlapping.

Posterior means of the parameters were obtained using a Gibbs sampler run for 20,000 iterations with a burn-in of 10,000 in the Holstein populations. For the Jersey population results were not consistent with only 20,000 iterations, so a chain length of 100,000 with a burn-in of 50,000 was used instead. The relatively poor mixing properties of the Gibbs sampler for the Jersey data could be due to the small size of the reference population. Lengths of the chains were based on preliminary runs and comparisons of the obtained segment variances. With 20.000 iterations the Holsteins showed a mean pairwise correlation between segment variances from 10 consecutive runs of 0.95, whereas the Jerseys showed a mean correlation between segment variances from 10 consecutive runs of 0.80. Increasing the number of iterations for the Jerseys to 100.0000 increased the mean correlation of segment variances between consecutive runs to .96.

### Prediction using location specific prior information

The purpose here is to build a Bayesian prediction model that allows for a larger proportion of variation to be explained by certain segments, based on knowledge from previous experiments. One way to do this is to allow different segments to have different prior probabilities assigned to the four SNP effect distributions. Letting ***S*** denote the set of segments, the model used here is:

(6) $$y=\mu 1+\sum_{s}W_{s}g_{s}+a+e$$

(7) $$g_{\mathrm{sj}}|\sigma_{\mathrm{sj}}^{2}\sim N\left(0,\sigma_{\mathrm{sj}}^{2}\right)\sigma_{\mathrm{sj}}^{2}=\{\begin{array}{c} 0*\sigma_{g}^{2}\;\text{with}\;\text{probability}\;\pi_{s0}\\ 0.0001*\sigma_{g}^{2}\;\text{with}\;\text{probability}\;\pi_{s1}\\ 0.001*\sigma_{g}^{2}\;\text{with}\;\text{probability}\;\pi_{s2}\\ 0.01*\sigma_{g}^{2}\;\text{with}\;\text{probability}\;\pi_{s3} \end{array}\;\sigma_{g}^{2}=r_{y}^{2}*\text{Var}\left(y\right)$$

(8) $$\pi_{s}\sim \text{Dirichlet}\left(\alpha_{s}\right)\;e\sim N\left(0,I\sigma_{e}^{2}\right)\;a\sim N\left(0,A\sigma_{a}^{2}\right)\sigma_{e}^{2},\sigma_{a}^{2},\mu \;\propto \;1$$

Here *π*_*s*_ is the probability vector for the four SNP effect distributions in segment s, and α_s_ is the vector of prior parameters for the Dirichlet distribution in segment s. The model is similar to the original BayesR model, with the modification that the probability to sample SNPs from the four different distributions now depends on the segment. By setting the location specific information via the Dirichlet prior, instead of using constant proportions, the model estimates the proportions using both the data and the prior information. As this is a BayesR by segment approach, the model will be referred to as BayesRS.

To test the BayesRS model, posterior means of the number of times in which the indicator variable fell in component *i* of the mixture were estimated in each segment in one population using BayesR, and subsequently used as the α_s_ parameters in the target population in BayesRS. This was done for segment sizes of 100, 250, 500, 1000, 2000 or 3000 SNPs. Since the sum of counts in the alpha parameters in this setup is equal to the number of markers, this means that the prior on the proportions in the mixture, unlike in the regular BayesR, now has as much weight as the data (much higher weight than in BayesR). To test the impact of the weight of the prior, different scaling factors were tried, i.e. the α_s_ parameters were multiplied by 0.2, 0.4, .0.6, 0.8, 1.0, 1.25 or 1.5. The model was tested in three different scenarios:

1. JER-AUS with prior information from HOL-AUS.

2. HOL-AUS with prior information from HOL-NOR.

3. HOL-AUS (random) with prior information from HOL-NOR.

HOL-AUS (random) is a random subset of 500 animals from the HOL-AUS reference population, which was generated to test the hypothesis that the advantage of the BayesRS model would be greater in smaller populations. The second and third setups were tested using the same validation animals.

### Validation of DGV accuracy

DGVs for the validation populations were predicted as

(9) $$DGV_{k}=\hat{\mu }+w{'}_{k}\hat{g}+{\hat{a}}_{k}$$

Where *w* ' _*k*_ is the row of **W** belonging to animal *k*. Accuracies of the DGV were calculated as r(DGV, DTD) and validated in HOL-AUS and JER-AUS. Differences in accuracy between BayesR and BayesRS were tested for significance using a Hotelling-Williams t-test, which takes account of the number of individuals in the validation set [15].

## Results and discussion

The posterior means of the number of times the indicator variable fell in each of the four distributions in BayesR for all three breeds and all three traits are shown in Table 2. Results are in line with [11] where it was also found that only a small percentage of the markers have an effect. The markers with the highest posterior probability of being in the largest effect distributions are unevenly distributed across the segments, and as the table also shows large proportions of the marker variance is expected to be explained by a small number of markers, which suggest that the location specific priors add extra information.

**Table 2:** Distribution of SNP effects and proportion of expected total marker variance for each class

	**JER-AUS**			**HOL-AUS**			**HOL-NOR**		
**Distribution**	**Protein**	**Fat**	**Milk**	**Protein**	**Fat**	**Milk**	**Protein**	**Fat**	**Milk**
0	462816 (0%)	461458 (0%)	461173 (0%)	462734 (0%)	461215 (0%)	462715 (0%)	460980 (0%)	461134 (0%)	460155 (0%)
0.0001	2413 (35%)	3752 (45%)	4111 (49%)	2623 (52%)	4223 (70%)	2487 (38%)	4387 (67%)	4270 (64%)	5205 (65%)
0.001	299 (44%)	318 (38%)	239 (28%)	179 (36%)	95 (16%)	332 (50%)	170 (26%)	127 (19%)	171 (21%)
0.01	14 (21%)	14 (17%)	19 (23%)	6 (12%)	9 (15%)	8 (12%)	5 (8%)	11 (17%)	11 (14%)

Posterior means of the number of times the indicator variable fell in each of the four distributions in the mixture for protein yield, fat yield and milk-yield in Australian Jersey (JER-AUS), Australian Holstein (HOL-AUS) and Nordic Holstein (HOL-NOR). Expected proportion of marker variance in each class was calculated as the number of markers in the class times the proportion of genetic variance assigned to each marker (0, 0.0001, 0.001 or 0.01) divided by the sum of marker variance in all classes.

### Segment variances

Table 3 shows the top 10 segments for all three traits and all three populations for a segment size of 100 SNPs. For all traits and populations there is one segment with very large effect, located on chromosome 14. This segment is located around the *DGAT1* gene which has a well-documented effect on the traits in question [16]. Furthermore for fat percentage in the Holsteins there is a segment with large effect on chromosome 5. This segment has previously been seen in GWAS studies in Australian Holsteins and validated in Australian Jerseys [17]. The segment, however, does not appear in the top 10 for the Jersey population. In the Nordic Holstein population a segment having large effect on milk yield is found on chromosome 20, but the same segment is not found in the top 10 for either JER-AUS or HOL-AUS although other segments on chromosome 20 are present. A previous study found a growth hormone receptor gene on chromosome 20 which was highly associated with milk yield [18]. However, the same gene could not be validated in the Australian Holstein population [19], and the segments on chromosome 20 found in the top 10 in this study do not coincide with the gene in question.

**Table 3:** Top 10 segments ranked by proportion of variance

**Protein yield**				**Fat yield**				**Milk yield**			
**Prop Var**	**CHR**	**Start**	**End**	**Prop Var**	**CHR**	**Start**	**End**	**Prop Var**	**CHR**	**Start**	**End**
**JER-AUS**											
**2.51**	**14**	**1324152**	**2524432**	**2.63**	**14**	**1324152**	**2524432**	**9.23**	**14**	**1324152**	**2524432**
0.94	8	53145498	53823453	0.53	6	32804873	33205790	1.03	23	33488986	34003600
0.49	29	32616370	33148216	0.5	16	34988324	35436695	0.88	20	17220850	17690967
0.46	13	63320438	64620664	0.26	9	47503904	48177473	0.75	16	35920	804371
0.38	23	36309465	36929103	0.21	27	35936818	36385854	0.45	23	39769575	40258674
0.34	3	89581663	89898693	0.2	1	127226200	127917611	0.33	10	30761581	31494927
0.31	17	69563223	70374082	0.18	22	60504152	60873210	0.24	8	73117701	73898467
0.31	29	33157623	33719571	0.17	12	34663468	35292842	**0.22**	**5**	**93922247**	**94302255**
0.3	29	34401183	34817726	0.15	13	27805120	28346480	0.2	20	28252035	28774228
0.25	9	48742490	49474068	0.15	5	120908187	121179132	**0.17**	**20**	**34452105**	**35077755**
**HOL-AUS**											
**10.38**	**14**	**1324152**	**2524432**	**17.97**	**14**	**1324152**	**2524432**	**16.05**	**14**	**1324152**	**2524432**
1.27	6	88537190	88996262	**2.71**	**5**	**93922247**	**94302255**	0.7	6	88537190	88996262
0.58	7	82862759	83380203	0.34	5	93301390	93920010	0.66	20	31054019	31704692
0.52	18	58283983	59602905	0.31	2	107799001	108408740	0.63	5	93301390	93920010
0.48	3	117198648	117541474	0.28	26	20643699	21338653	0.46	18	33639529	33910770
0.44	6	89469872	90304531	0.25	4	106613116	107060437	0.42	7	82862759	83380203
0.31	28	18048845	18758510	0.18	25	8073067	8481205	0.37	14	69793328	70364164
0.3	11	38575857	38932630	0.16	11	102944335	103540503	**0.33**	**20**	**34452105**	**35077755**
0.2	1	136016808	136624372	0.12	20	36176136	36613401	0.31	25	14686647	15151658
0.2	18	57084113	57818432	0.11	16	56014214	56706854	0.28	11	46783081	47198727
**HOL-NOR**											
**4.85**	**14**	**1324152**	**2524432**	**24.35**	**14**	**1324152**	**2524432**	**13.49**	**14**	**1324152**	**2524432**
0.39	19	26550090	27153052	**2.65**	**5**	**93922247**	**94302255**	2.37	20	29983162	31051302
0.31	7	23881292	24505374	0.76	19	20077363	20545023	0.9	5	92171816	92734379
0.31	5	20094983	20608440	0.64	5	20094983	20608440	0.89	5	92736297	93292054
0.28	6	86786552	87331055	0.5	15	44850860	45134081	0.63	5	20094983	20608440
0.25	11	102944335	103540503	0.26	20	63502967	63925075	**0.46**	**5**	**93922247**	**94302255**
0.23	22	42655109	43216893	0.25	19	26550090	27153052	0.38	15	52804974	53411913
0.18	23	11564383	12095383	0.23	2	127612583	128084951	0.33	24	59281770	59735242
0.16	24	59281770	59735242	0.21	26	20043160	20630551	0.33	6	88023038	88527916
0.16	20	69275055	69727331	0.19	13	10469479	11785572	0.23	11	101054186	101516564

Proportion of variance (Prop Var) explained for Jersey (JER-AUS), Australian Holstein (HOL-AUS) and Nordic Holstein (HOL-NOR) populations. Segment size is 100 SNPs. Start and end points of the segments are given as base positions of the first and last SNP in the segment. Segments that appear in more than one breed are highlighted in bold.

**Figure 1:**
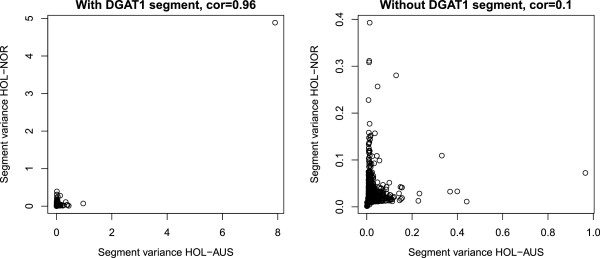
**Comparison of segment variances between Australian Holstein (HOL-AUS) and Nordic Holsteins (HOL-NOR) with and without the effect of *DGAT1*, shown here for protein yield and a segment size of 100 SNPs** .

**Figure 2:**
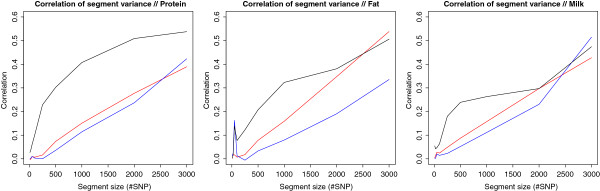
**Correlation of segment variances for Protein-, Fat- and Milk yield between JER-AUS and HOL-AUS (red), JER-AUS and HOL-NOR (blue) and HOL-AUS and HOL-NOR (black).** The segment containing *DGAT1* has been removed for all traits, as has the large effect on chromosome 5 for fat yield.

Correlations of segment variances between populations are large if the *DGAT1 *segment is included since the proportion of variance explained for this QTL is very large compared to all others, as seen from Table 3 and Figure 1. Hence SNPs associated with the *DGAT1* mutation have been removed from all plots in Figure 2 for clarity. For fat yield the QTL on chromosome 5 with large effect has been removed as well. Figure 2 shows a plot of segment size versus correlations of segment variances between the three populations for the three traits. With the large effect segments removed, the patterns of correlations generally follow expectations based on knowledge of genetic relationship between populations. Highest correlations are found between HOL-AUS and HOL-NOR, and second and third place are respectively HOL-AUS versus JER-AUS and HOL-NOR versus JER-AUS. In Australia some crossbreeding has taken place between the Holstein and Jersey populations and, accordingly, correlations of variance explained by the segments are higher for these two populations than between HOL-NOR and JER-AUS. Genotype by environment interactions could also influence these correlations.

**Figure 3:**
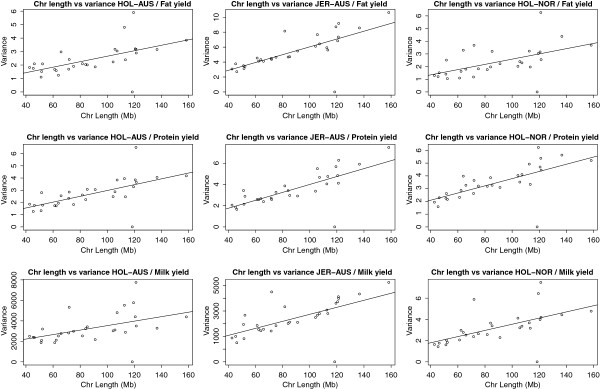
**Variance explained versus chromosome length for protein-, fat- and milk yield in the Jersey (JER-AUS), Australian Holstein (HOL-AUS) and Nordic Holstein (HOL-NOR) populations.** Chromosome 14 is not included in the plot.

Correlations for small segment sizes are close to zero. A possible explanation for this is that differences in LD patterns and SNP allele frequencies across breeds cause the SNPs with the highest associations to actual QTL to be placed in different segments when these are very small. The rapid increase in correlation of segment variances with segment size for HOL-AUS vs. HOL-NOR suggests that these population share QTL in similar locations, as would be expected given they are genetically closely related. For this pair of populations, even reasonably small segments would convey information between the populations. When taking an entire chromosome as a segment, correlations of segment variances ranging from 0.6 between JER-AUS and HOL-NOR up to 0.8 between the HOL-AUS and HOL-NOR were found. A connection between chromosome size and variance explained has previously been reported by e.g. [20]. As seen from Figure 3 a similar pattern can be found in our populations, provided the effect of *DGAT1* is removed from the analysis.

### BayesRS

**Figure 4:**
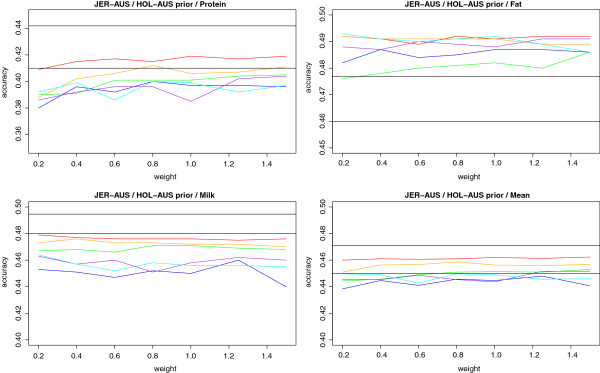
**Accuracy of DGV for Australian Jersey (JER-AUS) with prior Information from Australian Holstein (HOL-AUS).** Priors were tested for a segment sizes of 100 (red), 250 (orange), 500 (green), 1000 (blue), 2000 (purple) or 3000 (cyan) SNPs. X axis shows different weights on the prior information relative to the information from the data. Horizontal black lines are accuracies obtained using BayesR, where the higher lines gives the accuracy using pooled reference data from Jersey and Australian Holstein. No significant differences between accuracies obtained using BayesR with either a single or combined reference population and BayesRS were detected.

**Figure 5:**
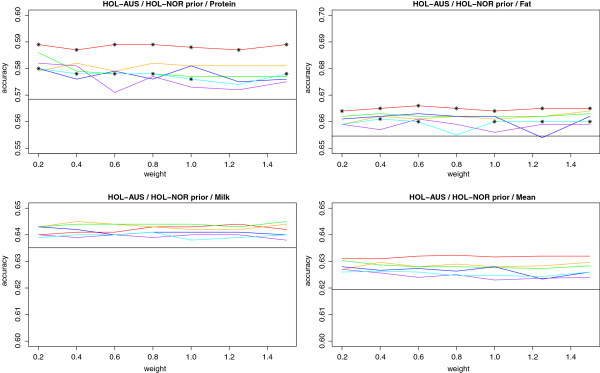
**Accuracy of DGV for Australian Holstein (HOL-AUS) with prior Information from Nordic Holstein (HOL-NOR).** Priors were tested for a segment sizes of 100 (red), 250 (orange), 500 (green), 1000 (blue), 2000 (purple) or 3000 (cyan) SNPs. X axis shows different weights on the prior information relative to the information from the data. Horizontal black lines are accuracies obtained using BayesR. BayesRS accuracies showing significant difference from the accuracy obtained using BayesR at a 5% significance level are marked with *. The test was only applied for single traits, and not for the mean.

The accuracies of DGV, measured as r(DGV,DTD) in the validation population, are shown in Figure 4 and 5. In the figures, a horizontal line depicts the accuracy using BayesR. For JER-AUS a second (higher) horizontal line shows the accuracies obtained from BayesR when simply pooling JER-AUS with HOL-AUS. For the HOL-AUS with HOL-NOR information scenario this accuracy was not available due to data sharing policies.

For JER-AUS no gain in accuracy was observed for milk yield when using prior information from HOL-AUS, for protein yield a small gain of around 1% is seen for the smallest segment size, and for fat yield gains in accuracy of up to 3.5% are seen when using the genomic location specific prior information compared to using BayesR. Compared with accuracies obtained with a simple pooling of reference data, the BayesRS approach leads to an extra gain of up to 1.5% for fat yield, but not for the other two traits. Although differences in accuracy were seen, none of the differences were significant at a 5% level, reflecting the small size of validation population.

**Figure 6:**
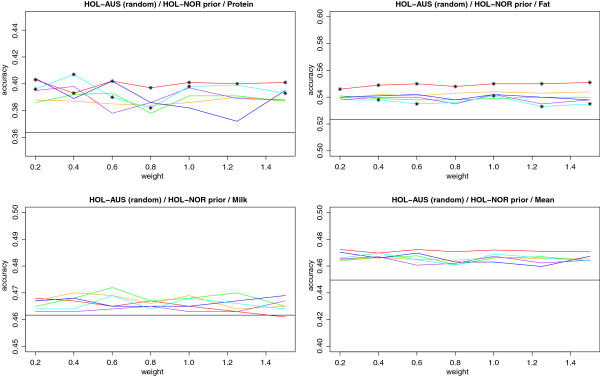
**Accuracy of DGV for a random subset of 500 Australian Holstein (HOL-AUS) bulls with prior Information from Nordic Holstein (HOL-NOR).** Priors were tested for a segment sizes of 100 (red), 250 (orange), 500 (green), 1000 (blue), 2000 (purple) or 3000 (cyan) SNPs. X axis shows different weights on the prior information relative to the information from the data. Horizontal black lines are accuracies obtained using BayesR. BayesRS accuracies showing significant difference from the accuracy obtained using BayesR at a 5% significance level are marked with *. The test was only applied for single traits, and not for the mean.

For HOL-AUS the largest gain in accuracy is found for protein yield with gains of up to 2%. For milk and fat yield smaller gains are seen, and these are not significantly different for milk yield. Using the prior information derived from HOL-NOR, however, seems consistently better than the model without location specific priors. Results from the HOL-AUS (random) setup are shown in Figure 6. The gain in accuracy is here slightly higher for protein and fat yield, whereas the gain for milk yield seems unchanged. Results were significant for protein and fat but not for milk yield. Looking at the accuracies of DGV from the regular BayesR model (no prior information from other populations) the mean accuracy is now on the same level as in JER-AUS due to the same size of the reference population (though the traits vary considerably).

In all three scenarios the highest gains in accuracy are found for a segment size of 100 markers, implying that using smaller segments gives a stronger advantage from the location specific priors. Furthermore, significant results are only found in two cases: the largest and smallest segments. For the largest segment size of 3000 markers, it is surprising that the increase in accuracy is significant although larger gains in accuracy are seen for smaller segments. However, this could be an artifact of the test chosen for the significance. With a large segment size the added information becomes very unspecific which could lead to results more similar to those obtained from the regular BayesR method. With a high correlation between DGVs from the two methods, the Hotelling Williams t-test would cause even small differences in accuracy to be significant.

The different scaling factors (weights) applied to the parameters in the Dirichlet priors, seems to make little or no difference on the accuracy of the BayesRS model, which suggests that the accuracies obtained from BayesRS could be random fluctuations. This is in many, but not all cases, supported by the lack of significance of the results.

To summarize, BayesRS gave accuracies comparable to, but not always higher than or significantly different from, a simple pooling of the data. For closely related populations pooling is expected to be superior. So a simple pooled multi-breed or multi-population reference could be a better approach in some cases, but not necessarily for all traits. For example, the BayesRS approach gave higher accuracies than a pooled reference for fat yield in JER-AUS. Further studies are needed to confirm the validity of the results in a larger validation population.

One advantage of the method presented here is a large reduction in computational demand. Since the BayesRS model only uses very condensed information from the other population, the increase in memory demand is negligible, and the extra complexity of the model only slightly increases the CPU run time. For JER-AUS running the BayesR model for 100.000 iterations required 33 hours, whereas the BayesRS model could be run for the same number of iterations in 39 hours. When using BayesR with the combined JER-AUS HOL-AUS reference data, 100,000 iterations takes about 150 hours, and more than quadruples the memory requirements.

Although the accuracies obtained using BayesRS in most cases cannot compete with pooling of the data, the results seem consistently better than when using only data from the target population and a non-informative prior, for example only the JER-AUS data. In some cases where the extra data itself is not available, the BayesRS model or a similar approach could improve the accuracy of genomic predictions using only summary statistics. This might be in cases when intellectual property issues prevents sharing of the raw data, but allows use of summary statistics as in this study. The approach could also be useful for meta-analysis of many data sets from different sources.

The model presented here would also allow the use of other prior information such as known QTL or expression pathways, by assigning a higher prior probability to sample large effects in the involved genomic regions. In this study segments were chosen arbitrarily with a fixed length, but another approach could be to define coding and non-coding regions of the genome as different segments and set different Dirichlet priors. A challenge here would, however, be how to choose the counts in the Dirichlet prior without sampling them from a different population. Previous results show that SNPs near genes found in both human and bovine genomes are significantly associated with stature [12]. By considering evolutionary conserved regions as segments the method using external information sources presented in this study could be used for genomic prediction across species for traits of common interest such as growth in meat-production animals or production traits in dairy species.

## Conclusion

Our results suggest that genomic location specific priors in BayesRS improve the accuracy of genomic prediction, when the priors are derived from another population. However, the higher accuracies were only found to be significantly better than a competing alternative without location specific priors in a few cases. This might be a result of the limited number of animals used in the validation sets, so further investigation is needed to confirm the validity of the method.

Results also show that some highly variable segments coincide with known genes and QTLs, suggesting that using actual biological information could be beneficial for the accuracy of genomic predictions. Finally the BayesRS setup might offer a possibility for higher accuracies of genomic predictions in cases with limited computer resources or issues with data sharing.

## Competing interests

The authors declare no competing interests.

## Authors’ contributions

Concept and design of the study was done by RFB, BJH, GS, MEG and MSL. The BayesRS model was developed by RFB, BJH and MEG. Implementation of statistical models in c++ was done by PJB. Analysis was carried out by RFB. The manuscript was drafted by RFB, BJH and GS. The final manuscript was read and approved by all authors.
